# Low-Cost and High-Strength Titanium–Zirconium–Oxygen Alloy Prepared by Spark Plasma Sintering

**DOI:** 10.3390/ma18225138

**Published:** 2025-11-12

**Authors:** Hongliang Xiang, Qinchang Wu, Weixuan You, Xiaoqiang Cai, Wei Zhao, Ye Huang, Xiangkai Zhang, Chaochao Wu

**Affiliations:** 1 School of Advanced Manufacturing, Fuzhou University, Jinjiang 362251, China; xhl@fzu.edu.cn (H.X.);; 2 School of Mechanical Engineering and Automation, Fuzhou University, Fuzhou 350108, China; zw@fdzcxy.edu.cn (W.Z.); cwu@fzu.edu.cn (C.W.)

**Keywords:** Titanium-zirconium-oxygen alloys, spark plasma sintering, microstructure, mechanical properties, strengthening mechanisms

## Abstract

Ti-Zr alloys are widely used in medical implants owing to their excellent biocompatibility. However, conventional alloying strategies to improve their performance often increase costs or introduce toxic elements. In this study, oxygen (O), a lightweight, cost-effective, and non-toxic element, was employed to strengthen Ti-Zr alloys. A novel Ti-Zr-O alloy was fabricated via spark plasma sintering (SPS), where the oxygen content was precisely controlled by incorporating TiO_2_ powder into the Ti-15Zr base powder. The sintered samples achieved a relative density above 99%, indicating nearly full densification under the optimized SPS conditions. Oxygen addition significantly refined the grain structure, while all O-containing samples maintained a uniform α-Ti phase with random crystal orientation. With increasing oxygen content, the compressive yield strength of the Ti-15Zr alloy increased from 619.24 MPa to 1634.18 MPa, accompanied by a decrease in compressive strain from 50.03% to 31.10%. These results demonstrate that the designed alloy combines superior yield strength with favorable ductility. Furthermore, quantitative analysis of the strengthening mechanisms revealed that oxygen atoms mainly occupy octahedral interstitial sites within the Ti-15Zr matrix, and solid-solution strengthening contributes more significantly than grain refinement. This work provides a promising route for the development of low-cost, high-performance Ti-Zr alloys for biomedical applications.

## 1. Introduction

Titanium–zirconium (Ti-Zr) alloys have emerged as preferred materials for medical implants owing to their outstanding mechanical properties and excellent biocompatibility [1,2,3,4]. However, the strength improvement achieved by simple binary Ti-Zr alloys remains limited [5]. To overcome this drawback, alloying with additional elements has been explored. For example, incorporating small amounts of Mo into Ti-Zr alloys suppresses grain growth, producing a refined microstructure that improves both strength and toughness [6]. Nevertheless, the continued addition of metallic elements may not only increase manufacturing costs but also introduce potential biotoxicity risks [7,8,9].

A promising strategy is to replace conventional alloying elements with lighter, more economical, and biocompatible alternatives. Oxygen (O), in particular, is known to significantly affect the mechanical behavior of titanium alloys. Ti-Zr alloys strengthened by O solid-solution, although fabricated via different techniques, have demonstrated enhanced strength and corrosion resistance while retaining favorable ductility and biocompatibility [10,11,12,13,14]. Liu et al. [15], for instance, developed high-oxygen-content (0.42–0.54 wt.%) Ti-5Zr, Ti-10Zr, and Ti-15Zr alloys by powder metallurgy and hot working. Among them, Ti-15Zr achieved a tensile strength of 914.2 MPa, yield strength of 891.2 MPa, and elongation of 20.2%, indicating an excellent balance of strength and ductility. Oxygen may originate from raw powders or be introduced during mixing and sintering, and its content can usually be precisely controlled by adding TiO_2_ [16] or ZrO_2_ [17]. Overall, research on biomedical Ti-Zr alloys has largely focused on improving mechanical properties and production efficiency while maintaining biocompatibility [18].

Despite these advances, conventional fabrication techniques such as arc melting [19,20,21,22] and powder metallurgy methods—including hot pressing, hot isostatic pressing, pressureless sintering, and high-pressure sintering [23,24]—still face limitations in the development of biomedical Ti-Zr alloys. Arc melting is energy-intensive, prone to compositional segregation, and often results in ingots with poor workability due to the hexagonal close-packed (HCP) structure of titanium alloys at room temperature [25,26,27]. Traditional powder sintering, on the other hand, suffers from relatively low efficiency: prolonged exposure to high temperatures can lead to grain coarsening [28,29], whereas insufficient temperature or pressure results in poor densification and high porosity [30]. Consequently, it is difficult to achieve a satisfactory balance between strength and ductility using these approaches.

Spark plasma sintering (SPS), an emerging powder metallurgy technique, offers unique advantages for fabricating dense and high-performance materials. Its rapid heating and cooling rates enable efficient densification while minimizing grain coarsening [31,32]. Compared with conventional powder metallurgy processes, SPS provides superior production efficiency and material utilization, making it especially suitable for preparing small-batch, high-performance titanium implants [33]. This has been demonstrated in alloys such as Ti-Nb [34], Ti-6Al-4V [35], Ti-Nb-Zr [36], and Ti-Nb-Sn [37]. However, the application of SPS to the preparation of Ti-Zr-O alloys has not yet been fully explored. While SPS offers clear advantages, other emerging techniques such as microwave sintering are also being explored for advanced material processing [38]. However, Microwave sintering, in particular, enables rapid and energy-efficient heating, achieving high densification at relatively low temperatures through the interaction between electromagnetic fields and materials. Its effectiveness strongly depends on the dielectric properties of the material, which makes it difficult to uniformly heat highly conductive metals such as titanium alloys, often resulting in localized overheating or nonuniform densification. The unique combination of rapid heating and simultaneous pressure in SPS is particularly effective for achieving full density while retaining fine-grained microstructures, making it highly suitable for the goals of this study.

In this study, we aim to develop cost-effective and high-strength Ti-Zr-O alloys using SPS. The microstructure, mechanical properties, and strengthening mechanisms are systematically investigated. By optimizing the SPS parameters and precisely controlling the O content, this work demonstrates the potential of SPS for fabricating advanced Ti-Zr-based biomedical alloys with enhanced strength and toughness while ensuring reliable biocompatibility. The findings are expected to provide both theoretical insights and a technical foundation for further application of Ti-Zr alloys in the biomedical field.

## 2. Experimental Methods

### Materials Preparation

**Figure 1:**
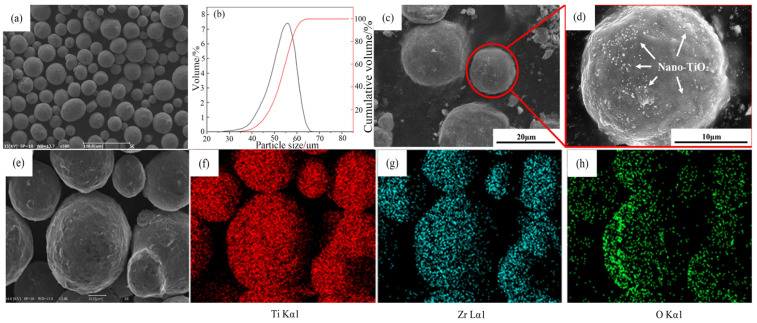
(**a**) SEM morphology of the composite powders after mixing for 3 h in a V-type mixer; (**b**) Particle size distribution of the composite powders; (**c**,**d**) Nano-TiO_2_ particles dispersed on the surface of the spherical Ti-15Zr powder; (**e**–**h**) Corresponding EDS elemental maps of Ti, Zr, and O for the area in (**e**), confirming the distribution characteristics of the elements.

The Ti-15Zr pre-alloyed powder was produced by electrode induction melting and gas atomization, and its chemical composition is listed in Table 1. The SEM image of the Ti-15Zr base powder (Figure 1a) shows that the particles are predominantly spherical with smooth surfaces and high sphericity. The particle size distribution, measured using a BT-9300S laser particle size analyzer (Dandong City, China), follows an approximately log-normal distribution (Figure 1b). The particle size ranges from 10 μm to 55 μm, with D10 = 12.00 μm, D50 = 27.95 μm, and D90 = 55.33 μm. The TiO_2_ powder, used as the oxygen source, consists of layered nanoscale particles with an average size of 20 nm and a purity of 99.99%.

The Ti-15Zr base powder and TiO_2_ powder were dried in a vacuum oven at 100 °C for 2 h to remove adsorbed moisture. The two powders were then mixed in a V-blender under vacuum conditions for 3 h. Figure 1c shows the surface morphology of the Ti-15Zr powder after mixing, and Figure 1e–h presents the corresponding EDS elemental mapping. The results indicate that the nanoscale TiO_2_ powder did not exhibit aggregation on the Ti-15Zr particle surfaces but was uniformly distributed across the spherical powder. Such homogeneous distribution is beneficial for achieving uniform dispersion and dissolution of TiO_2_ in the matrix during the subsequent SPS process. By adjusting the amount of TiO_2_ powder added, Ti-15Zr alloy powders with different oxygen contents were successfully obtained.

**Figure 2:**
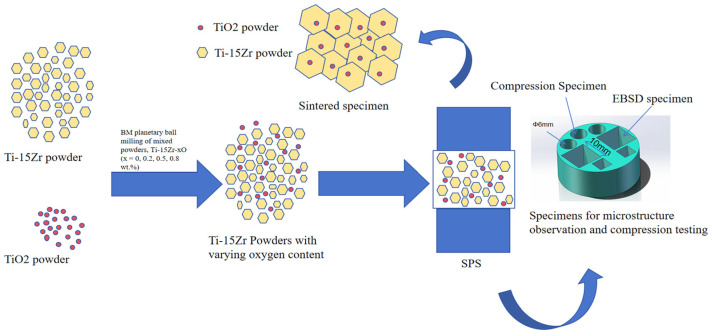
Schematic diagram of SPS process.

Figure 2 illustrates the overall SPS process. The sintering was performed using the LABOX-1575 SPS equipment produced by Sinter Land Co., Nagaoka City, Japan under the following processing parameters in Table 2:

The SPS process produced sintered samples with a diameter of 30 mm and a thickness of 12 mm. Microstructural specimens with dimensions of 10 mm × 10 mm × 10 mm were sectioned from the bulk samples, ground with 2000-grit sandpaper, and subsequently electropolished in an electrolyte of 10% perchloric acid in anhydrous methanol. The actual oxygen contents of the Ti-15Zr samples with different TiO_2_ additions were measured by the inert gas fusion method. Phase identification was performed using X-ray diffraction (XRD, DY5261, CEM, Matthews, NC, USA) with Cu-Kα radiation. Microstructural characterization was carried out by optical microscopy (OM, MV5000, Olympus Corporation, Tokyo, Japan) and scanning electron microscopy (SEM, Regulus 8100, Hitachi). Grain size and orientation distributions were analyzed by electron backscatter diffraction (EBSD, Symmetry S2, Oxford Instruments, Abingdon, UK). Cylindrical specimens (Φ6 mm × 10 mm) were used for compression testing on a universal testing machine (E45.105, MTS Systems Co., Ltd., Eden Prairie, MN, USA). Fracture surface morphologies were further examined using SEM (Quanta 400, Raleigh, NC, USA).

## 3. Results and Discussion

### 3.1. Densification Behaviors of Ti-15Zr

**Figure 3:**
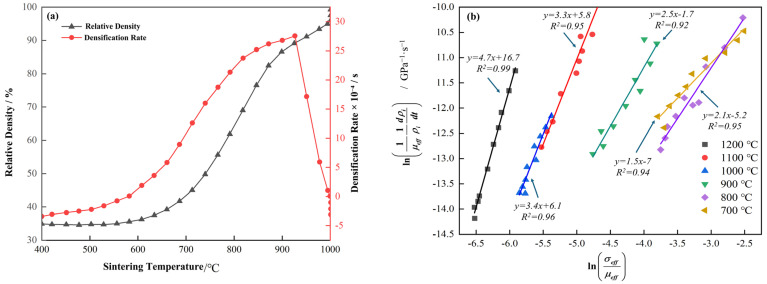
(**a**) Curves showing the variation in density and densification rate with temperature at a sintering temperature of 1000 °C; (**b**) Fitting of the effective stress exponent n at different sintering temperatures during the SPS process according to Equation (1).

In this section, the processing parameters for sintered specimens are optimized to achieve maximum densification. Sintering temperature is a critical factor influencing both the density and the forming quality of the specimens. As a representative case, Figure 3a shows the curves of density and densification rate as functions of temperature. During the heating stage of the SPS process, the densification rate of the sintered specimens increases significantly when the sintering temperature ranges from 600 °C to 900 °C. Notably, the densification rate reaches a maximum at 926 °C. However, further temperature increases result in a decline in the densification rate. It is worth noting that the temperature corresponding to the maximum densification rate (926 °C) is slightly higher than the phase transformation temperature of the Ti-15Zr alloy (889 °C) [1]. Moreover, as the density has already attained a high level (99.29% at 900 °C), further enhancement in the densification rate becomes increasingly difficult.

To gain deeper insight into densification behavior at different sintering temperatures, a series of equations can be derived to determine and fit the stress exponent factor n in the SPS process, yielding the following Equation [39]:

(1) $$\ln\left(\frac{1}{\mu_{eff}}\frac{1}{\rho_{i}}\frac{d\rho_{i}}{dt}\right)=n\ln\left(\frac{\sigma_{eff}}{\mu_{eff}}\right)+K_{1}$$

where $\mu_{eff}$ represents the instantaneous effective shear modulus, $\sigma_{eff}$ is the instantaneous effective stress applied to the powder, $K_{1}=\ln\left[Bb\Phi_{0}/k\right]$ is a constant, where $\Phi_{0}$ is the pre-exponential factor of the diffusion coefficient, Φ and B are constant depending on the sintering material, b is the Burgers vector, and k is the Boltzmann constant. For a given holding temperature, $K_{1}$ can be regarded as a constant; $\rho_{i}$ represents the instantaneous relative density. For Equation (1), it can be viewed as a linear function of $\ln\left[\left(1/\mu_{eff})\cdot (1/\rho_{i})\cdot (d\rho_{i}/dt\right)\right]$ and $\ln\left(\sigma_{eff}/\mu_{eff}\right)$, where the slope of this linear function is n. The results are illustrated in Figure 3b. Specifically, the stress exponent factor n displays a gradual increasing trend within the temperature range of 700 °C to 1200 °C, with specific values as follows: 1.5, 2.1, 2.5, 3.4, 3.3, and 4.7. These changes reflect a transition in densification mechanisms at different temperatures. When n≤2, it suggests that, under certain sintering temperatures, densification primarily occurs through a pure diffusion mechanism similar to volume diffusion [40]. In contrast, when n falls between 3 and 5, particularly under higher sintering temperatures, the densification mechanism is associated with high-temperature creep (dislocation climb) [41,42]. It can be deduced that at 700 °C, the densification process is primarily governed by a pure diffusion mechanism, i.e., atomic diffusion driven by thermal motion is the main force promoting densification. As the temperature rises to 800 °C and 900 °C, the densification mechanism shifts to a combination of diffusion and high-temperature creep. This transition suggests that with further temperature increases, the contributions of thermal activation and creep mechanisms to densification begin to intensify. Within the 1000 °C to 1200 °C range, densification is predominantly controlled by high-temperature creep, indicating that at higher temperatures, the creep mechanism encompassing dislocation motion and diffusion creep becomes the primary driving force for densification. This mechanism significantly impacts the material’s microstructure and macroscopic properties.

### 3.2. Microstructure

**Figure 4:**
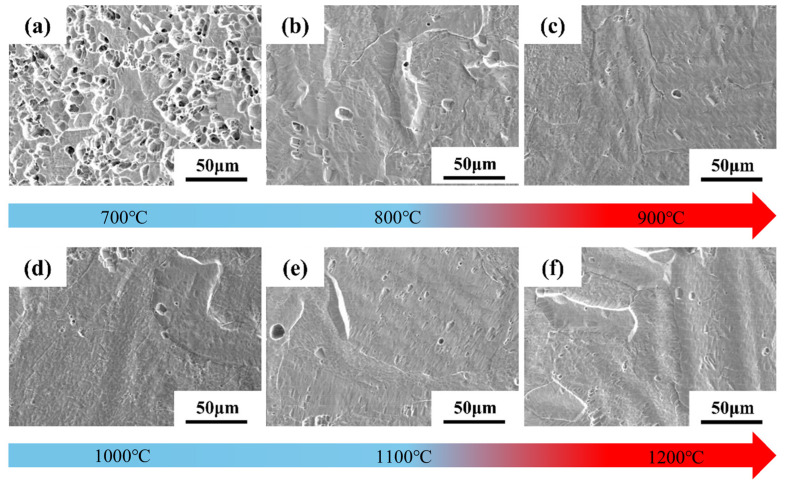
SEM images of the microstructural surface morphology of Ti-15Zr sintered specimens under sintering temperatures of 700–1200 °C: (**a**) 700 °C; (**b**) 800 °C; (**c**) 900 °C; (**d**) 1000 °C; (**e**) 1100 °C; (**f**) 1200 °C.

Figure 4 shows SEM images of the microstructural surface morphology of Ti-15Zr sintered specimens at sintering temperatures ranging from 700 °C to 1200 °C. As illustrated in Figure 4a, at 700 °C, the specimens exhibit numerous pore defects, and the boundaries between particles are clearly discernible. This suggests that, at lower sintering temperatures, although softening and plastic deformation between particles have begun, both the connectivity and the number of pores remain relatively high. As the sintering temperature increases, particularly at 900 °C, porosity is significantly reduced. While some pores remain, the boundaries of the original powder particles become increasingly indistinct, indicating that the specimen has achieved near-complete densification. Figure 4d–f show that, within the 1000 °C to 1200 °C range, densification continues at a slower rate, with only minimal changes in pore quantity and morphology.

Since the atomic arrangement in the HCP structure of α-Ti is more compact than that of the BCC structure of β-Ti, the self-diffusion rate of β-Ti above the phase transformation temperature is approximately 100 times higher than that of α-Ti. This accelerated diffusion promotes grain growth and grain boundary migration, thereby significantly enhancing material densification [43]. Consequently, during sintering above the phase transformation temperature, β-Ti—owing to its superior self-diffusion and plastic deformation capabilities—exhibits a markedly faster densification rate compared to α-Ti with an HCP structure. In summary, selecting a sintering temperature between 1000 °C and 1200 °C ensures that the unique properties of β-Ti are fully exploited during the sintering process, yielding highly dense specimens.

To prepare Ti-15Zr alloys with varying oxygen contents, different mass fractions of TiO_2_ powder (0, 0.50, 1.25, and 2.00 wt.%) were added to the initial Ti-15Zr matrix powder, which had a base oxygen content of 0.12 wt.%. Table 3 presents the actual oxygen content measured in the composite powders after mixing and in the final samples after sintering. The results confirm a slight oxygen increase during the sintering process. For ease of reference, the sintered samples are designated by their nominal oxygen content; for instance, the sample corresponding to the addition of 1.25 wt.% TiO_2_ is labeled as 0.5O.

**Figure 5:**
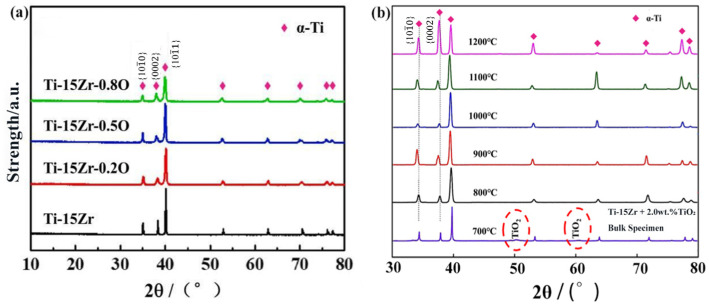
(**a**) XRD patterns of sintered Ti-15Zr-xO samples at 1000 °C (x = 0, 0.2, 0.5, 0.8 wt.%); (**b**) XRD of Ti-15Zr-0.8O material at different sintering temperatures. Red circles mark TiO_2_ peaks.

The XRD patterns of sintered Ti-15Zr-xO (x = 0, 0.2, 0.5, 0.8 wt.%) samples at 1000 °C are shown in Figure 5. The results indicate that only the characteristic peaks of α-Ti with an HCP structure are observed in all sintered samples, with no detectable peaks from other compounds, including TiO_2_. This finding agrees with previous studies on the decomposition of titanium oxides [44], which suggest that at temperatures below 400 °C, TiO_2_ tends to decompose into products such as Ti_3_O_5_, Ti_2_O_3_, TiO, and Ti. Furthermore, at temperatures above 400 °C, TiO_2_, TiO, and other sub-oxides continue to decompose, with their components diffusing into the titanium matrix. Driven by concentration gradients, oxygen atoms migrate from TiO_2_ into the Ti-15Zr matrix. During the SPS process, plasma primarily forms around the contact points between powder particles, resulting in the actual temperature at the sintering neck being higher than the bulk sintering temperature, thereby providing additional energy to facilitate diffusion [45]. These effects collectively indicate that oxygen atoms, acting as solute atoms, are successfully dissolved in the α-Ti matrix.

The analysis of Figure 5b further demonstrates that at a sintering temperature of 700 °C, the characteristic peaks of TiO_2_ are significantly weakened, suggesting that the decomposition process has begun but is not yet fully completed. When the sintering temperature exceeds 900 °C, the characteristic peaks of TiO_2_ disappear entirely, indicating that the decomposition reaction has been fully accomplished.

Similar observations have also been reported in the literature. Cai et al. [46] investigated Ti–O alloys fabricated by powder metallurgy and found that when the nominal oxygen addition was 1.5 at.% (actual oxygen content ≈ 1.8 at.%) at sintering temperatures of 1000 for 1 h under an axial pressure of 30 MPa, only Ti peaks were detected in the XRD patterns. This indicates that TiO_2_ particles were completely decomposed and the oxygen atoms were fully dissolved into the Ti matrix under such conditions. Their results strongly support the assumption that TiO_2_ can be fully decomposed during high-temperature SPS processing, leading to complete oxygen dissolution in the present study as well.

**Figure 6:**
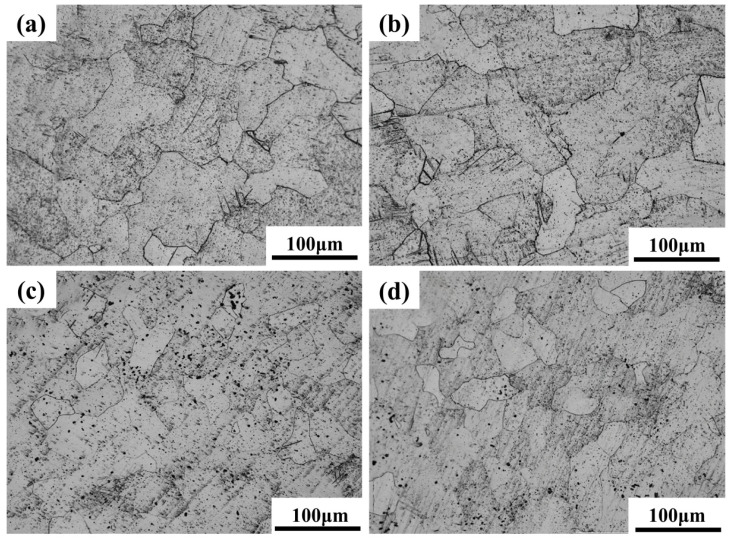
OM images of sintered Ti-15Zr-xO samples at 1000 °C: (**a**) x = 0 wt.%; (**b**) x = 0.2 wt.%; (**c**) x = 0.5 wt.%; (**d**) x = 0.8 wt.%.

**Figure 7:**
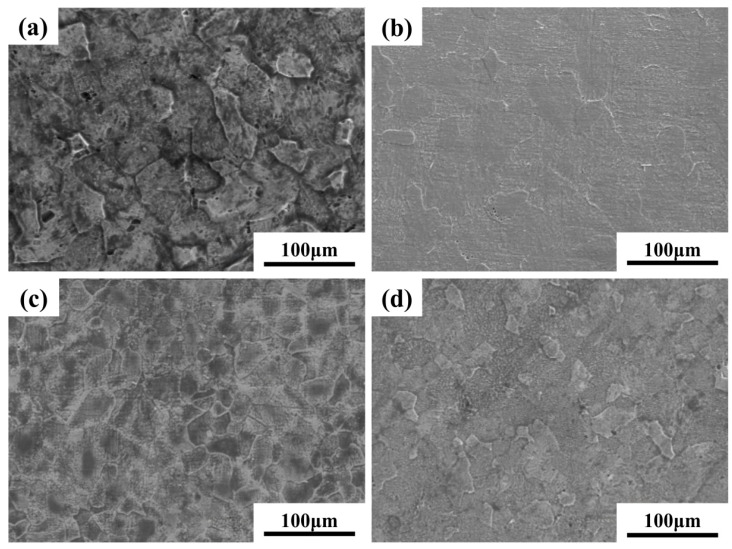
SEM images of sintered Ti-15Zr-xO samples at 1000 °C: (**a**) x = 0 wt.%; (**b**) x = 0.2 wt.%; (**c**) x = 0.5 wt.%; (**d**) x = 0.8 wt.%.

Figure 6 and Figure 7 present the optical microscopy (OM) and scanning electron microscopy (SEM) images of Ti-15Zr-xO sintered samples at 1000 °C. As observed in the XRD results, all sintered samples exhibit the typical characteristics of the α-phase structure, with no evidence of TiO_2_ phase separation or residual TiO_2_ particles. This suggests that the introduced TiO_2_ was effectively decomposed and its components were uniformly distributed within the alloy matrix. With the gradual increase in oxygen content, a clear trend of grain size reduction can be observed in the samples, as shown in Figure 6. Notably, when the O content reaches 0.8 wt.%, the reduction in grain size becomes more significant. This result demonstrates that the addition of TiO_2_ contributes to grain refinement in the sintered specimens.

Moreover, both OM and SEM images reveal the presence of porosity defects in the sintered samples. Such pores may affect the mechanical properties and density of the material. However, SEM observations show that the number of pores decreases markedly as the O content increases. This reduction can be attributed to the role of oxygen as an interstitial solute, which dissolves into the α-Ti matrix and accelerates atomic diffusion at grain boundaries, thereby facilitating effective particle bonding and reducing pore formation. Consequently, the porosity of SPS-sintered samples is significantly lower than that of conventional powder metallurgy products. Furthermore, density measurements using the Archimedes drainage method confirm that all sintered samples achieve a relative density exceeding 99%. These results verify that SPS-processed samples possess high overall density and compactness without requiring additional hot pressing, which in turn contributes to the enhancement of the mechanical properties of Ti-Zr alloys.

**Figure 8:**
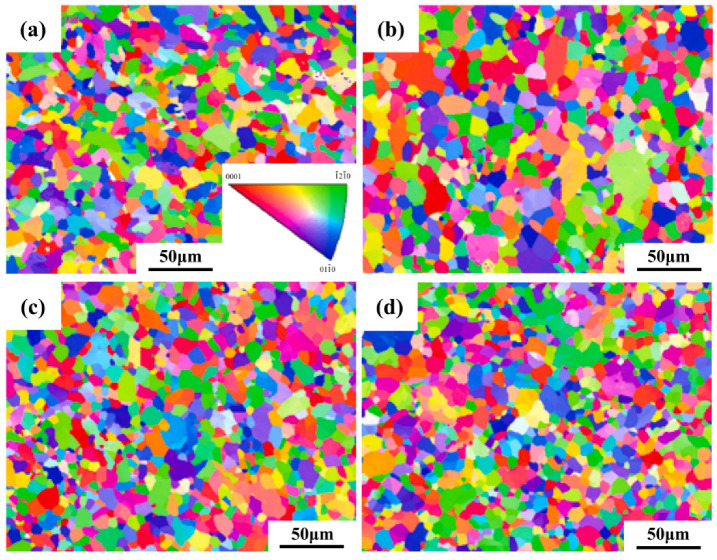
IPF maps of sintered Ti-15Zr-xO samples at 1000 °C (x = 0, 0.2, 0.5, 0.8 wt.%): (**a**) x = 0 wt.%; (**b**) x = 0.2 wt.%; (**c**) x = 0.5 wt.%; (**d**) x = 0.8 wt.%.

Figure 8 presents the inverse pole figure (IPF) analysis obtained from electron backscatter diffraction (EBSD) for the Ti-15Zr-xO sintered samples at 1000 °C. It can be observed that all Ti-15Zr-xO sintered samples exhibit equiaxed α-Ti grains, with no evidence of needle-like structures. This result demonstrates that the addition of oxygen does not alter the fundamental grain morphology of the alloy. Moreover, as the concentration of dissolved oxygen increases, EBSD analysis reveals that the distribution of crystal orientations remains uniform across all sintered samples, and no pronounced substrate texture is detected. These findings confirm that a uniform crystallographic orientation is preserved even with significant oxygen additions, without inducing the formation of texture.

**Figure 9:**
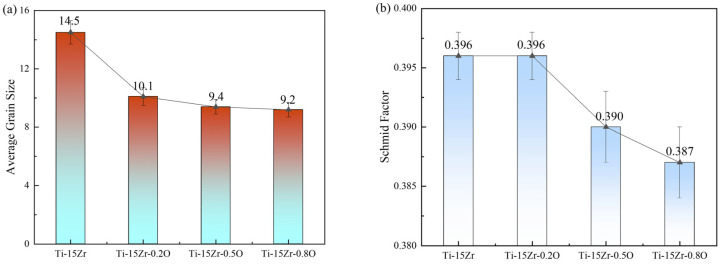
Statistical analysis of microstructural and crystallographic features for the as-sintered Ti-15Zr-xO alloys: (**a**) The variation in average grain size; (**b**) The corresponding average Schmid factor for each alloy composition.

Figure 9a presents the average grain size data of the Ti-15Zr-xO sintered samples at 1000 °C measured by EBSD. The data shows that as the oxygen content increases, the average grain size of the sintered samples decreases from Specifically, the average grain size decreases from 14.5 ± 0.8 μm for Ti-15Zr (0.14 wt.% O) to 10.1 ± 0.6 μm for Ti-15Zr-0.20, 9.4 ± 0.5 μm for Ti-15Zr-0.5 O, and 9.2 ± 0.5 μm for Ti-15Zr-0.8 O. This demonstrates that the addition of TiO_2_ effectively suppresses grain growth during sintering, leading to significant microstructural refinement.

In Figure 9b, the Schmid factor of the Ti-15Zr-xO sintered samples gradually decreases from 0.396 ± 0.002 (Ti-15Zr and Ti-15Zr-0.2 O) to 0.390 ± 0.003 (Ti-15Zr-0.5 O) and 0.387 ± 0.003 (Ti-15Zr-0.8 O). However, the small magnitude of this variation falls within the confidence interval, indicating that the difference is not statistically significant. Although a slightly lower average Schmid factor could imply less favorable orientation of the primary slip systems and hence higher resistance to dislocation motion, this contribution is likely negligible compared to oxygen solid-solution strengthening and grain refinement effects, which dominate the observed strength improvement.

### 3.3. Mechanical Properties

**Figure 10:**
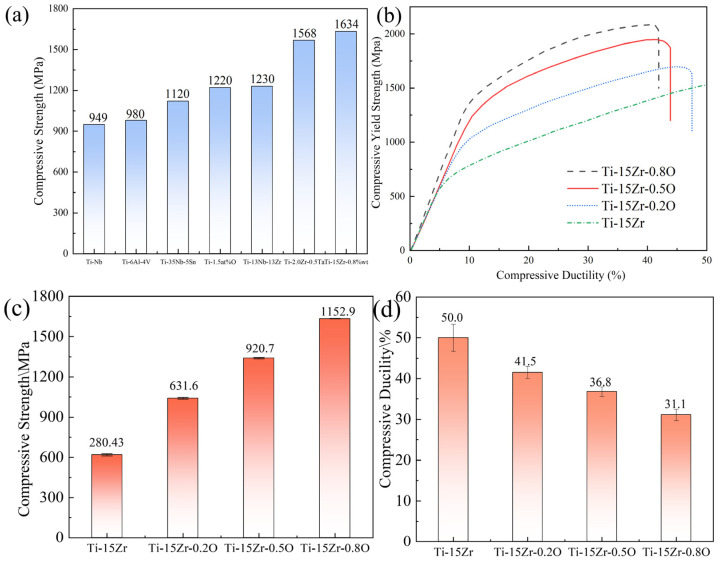
Compressive mechanical properties of as-sintered Ti-15Zr-xO alloys: (**a**) Comparison of compressive yield strength with other titanium alloys; (**b**) Representative compressive engineering stress–strain curves; (**c**) Compressive yield strength as a function of oxygen content; (**d**) Compressive ductility as a function of oxygen content.

Figure 10a compares the compressive strength of the Ti-15Zr-0.8O sintered specimen at 1000 °C with that of Ti-Nb [34], Ti–35Nb–5Sn [47], Ti-6Al-4V [48], Ti-2.0Zr-0.5Ta [49], commercially pure titanium containing 1.5 wt.% oxygen [46] and the well-established Ti-13Nb-13Zr biomedical alloy [50], while Table 4 summarizes the detailed compressive mechanical properties. The Ti-15Zr-0.8O specimen exhibits significantly higher strength than both reference materials. Figure 10b shows the stress–strain curves of the Ti-15Zr-xO sintered specimens, while Figure 10c,d highlights the compressive yield strength and strain behavior. The oxygen-free Ti-15Zr specimen demonstrates a yield strength of 619.24 MPa without fracturing even at compressive strains exceeding 40%. Upon oxygen addition, the mechanical performance improves markedly. The Ti-15Zr-0.2O specimen achieves a yield strength of 1040.10 MPa with a compressive strain of 41.52%, surpassing most reported biomedical titanium alloys. At higher oxygen contents, Ti-15Zr-0.5O reaches a yield strength of 1340.73 MPa with a strain of 36.86%, maintaining a favorable balance between strength and ductility. Notably, Ti-15Zr-0.8O continues to show an increasing trend in yield strength, although with a reduction in compressive strain. The overall mechanical property data, including density, yield strength, and compressive strain, are summarized in Table 5. These results reveal a nearly linear increase in strength with oxygen concentration, confirming oxygen as an effective solid-solution strengthener for Ti-Zr alloys. It is important to address the strength–ductility trade-off inherent in this alloy system. While the compressive strain decreases from 50.0% to 31.1% as the yield strength increases to 1634.1 MPa, it is critical to note that a compressive strain of over 30% is still considered substantial and indicates favorable ductility.

As shown in Figure 10d, the compressive strain of the Ti-15Zr-xO alloys gradually decreases from 50.0% to 31.1% as the oxygen content increases from 0.14 wt.% to 0.83 wt.%. This decline indicates that oxygen addition significantly enhances lattice friction stress and restricts dislocation mobility, leading to reduced compressive ductility. However, within the investigated range, the alloy does not reach an “unfeasible limit” where the material becomes excessively brittle. Even at the highest oxygen concentration (0.83 wt.%), the Ti-15Zr-O alloy still exhibits more than 30% compressive strain, which reflects a favorable balance between strength and deformability. Comparable results have been reported for oxygen-strengthened titanium alloys fabricated by powder metallurgy and spark plasma sintering. For instance, Liu et al. [51] observed that Ti–15Zr–O alloys with oxygen contents up to 0.8 wt.% exhibited compressive strains above 30%, while further increasing oxygen above 1.0 wt.% led to a rapid loss of plasticity. Similarly, Yamamoto et al. [52] found that oxygen-induced solid-solution strengthening in Ti–O systems increases yield strength significantly, but compressive strain drops sharply once oxygen exceeds ~1.0 wt.%. Therefore, the present results suggest that the critical oxygen concentration marking the transition to unfeasible brittleness likely lies beyond 0.8–1.0 wt.% O. The Ti-15Zr-O alloys prepared in this work (≤0.83 wt.% O) thus maintain an optimal compromise between high strength (up to 1634 MPa) and sufficient compressive ductility, confirming that controlled oxygen solid-solution strengthening can be effectively achieved without embrittlement.

This trade-off between strength and ductility is a classic manifestation of solid-solution strengthening. The reduction in plasticity with increasing oxygen content can be attributed to the strong hindrance of dislocation motion. As interstitial solute atoms, oxygen occupies the octahedral interstitial sites of the α-Ti lattice, producing significant local lattice distortions. These distortions generate strong stress fields that obstruct dislocation glide. Moreover, the elastic interactions between oxygen atoms and dislocations result in a pronounced pinning effect, raising the critical resolved shear stress required for slip initiation and propagation. Consequently, higher oxygen concentrations lead to greater resistance against dislocation motion, making plastic deformation progressively more difficult and ultimately lowering the compressive strain before fracture.

It is acknowledged that for long-term biomedical applications, particularly in dynamic, load-bearing scenarios, fatigue resistance is a critical performance metric. This study focused on the quasi-static compressive properties to establish a foundational understanding of the structure–property relationships and to identify the most promising compositions from a strength and ductility perspective. A comprehensive investigation into the fatigue life, crack initiation, and propagation behavior of these high-strength Ti-Zr-O alloys is, therefore, a crucial next step in our ongoing research. Such studies will be essential to fully validate their suitability for dynamic, load-bearing applications.

Regarding the chemical stability for biomedical applications, both titanium and zirconium are renowned for their excellent biocompatibility and corrosion resistance. This is primarily attributed to the spontaneous formation of a highly stable, inert, and strongly adherent passive oxide layer (composed mainly of TiO_2_ and ZrO_2_) on their surfaces in physiological environments [53]. In fact, Ti-Zr alloys are often reported to exhibit even superior corrosion resistance and biocompatibility compared to pure Ti, making them highly suitable for long-term implantation [54]. The element oxygen, added as an interstitial solute in this study, is not only non-toxic but is a fundamental component of this protective surface film. Therefore, it is anticipated that the developed Ti-Zr-O alloy will exhibit excellent chemical stability and minimize the risk of ion release. However, to definitively validate its long-term performance in vivo, comprehensive in vitro studies, such as electrochemical corrosion tests in simulated body fluid (SBF) and cytotoxicity assessments, are required. These investigations form a critical part of our future work to confirm the suitability of this material for clinical applications.

### 3.4. Strengthening Mechanisms

Before quantitatively analyzing the strengthening contributions, it is crucial to understand why the SPS-processed alloys in this study exhibit strengths far superior to their conventionally processed counterparts. For example, a Ti-15Zr alloy produced by traditional casting and forging has a reported yield strength of approximately 820 MPa [55], whereas the baseline Ti-15Zr alloy in this work already reaches 619.24 MPa, with the oxygen-alloyed version achieving an exceptional 1634 MPa. This significant deviation is fundamentally attributed to the unique microstructural features imparted by the Spark Plasma Sintering (SPS) technique.

**Figure 11:**
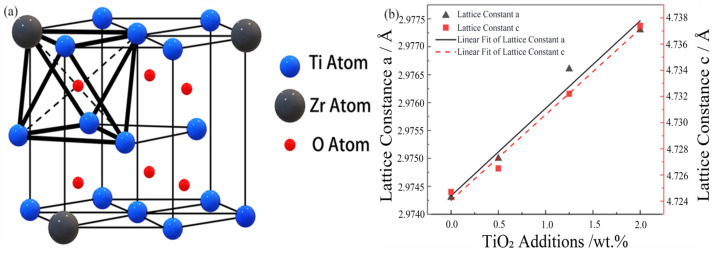
(**a**) Schematic diagram of Ti-15Zr-O alloy; (**b**) The *X*-axis represents the nominal weight percent of TiO_2_ added to the base powder. The corresponding measured oxygen contents are 0.14, 0.26, 0.55, and 0.83 wt.%, respectively.

A fundamental aspect of these microstructural features is the manner in which oxygen is incorporated into the host lattice, which can be elucidated through crystallographic analysis. To this end, an analysis of the XRD data shown in Figure 5 was conducted. It was observed that with the gradual increase in oxygen content, the diffraction peaks of α-Ti at the ($00\bar{1}0$) and (0002) crystal planes shifted to lower diffraction angles. This phenomenon indicates that the dissolved oxygen atoms caused an increase in the lattice constants along both the a-axis and c-axis, as illustrated in Figure 11a. In the undoped Ti-15Zr alloy, the lattice constants a and c were measured to be 2.9743 Å and 4.7247 Å, respectively, as shown in Figure 11b. With the increase in the amount of TiO_2_ added, the lattice constant c increased from 4.7247 Å to 4.7374 Å, while the lattice constant a increased from 2.97433 Å to 2.9769 Å. This change resulted in an increase in the c/a ratio from 1.5885 to 1.5914. The preferential occupation site of interstitial atoms can be inferred by the nature of the lattice distortion they induce; theoretical models predict that occupation of octahedral sites in α-Ti leads to a more significant expansion along the c-axis and thus an increase in the c/a ratio. Based on these results, it can be concluded that oxygen atoms primarily occupy octahedral interstitial sites within the α-Ti matrix. This finding aligns with the predictions made by Scotti et al. [56], indicating that the interstitial diffusion of oxygen atoms in the octahedral sites has the lowest interstitial energy.

The rapid, non-equilibrium nature of SPS provides two key advantages over traditional methods. Firstly, it produces an ultra-fine grain structure (9.2–14.5 μm) that provides a significant Hall–Petch strengthening contribution, which is less pronounced in coarse-grained cast materials. Secondly, it facilitates a high degree of uniform, supersaturated solid solution of oxygen atoms within the α-Ti lattice, which maximizes the potent interstitial strengthening effect. Therefore, the remarkable strength of these alloys is a direct result of their advanced, non-equilibrium microstructure. The following analysis will quantitatively deconstruct the contributions from these mechanisms.

In this study, two primary strengthening mechanisms are considered: solid-solution strengthening due to dissolved oxygen atoms and grain refinement strengthening, also known as Hall–Petch strengthening. As interstitial atoms, oxygen occupies octahedral interstitial sites [57], generating a pronounced pinning effect on dislocation motion and effectively impeding dislocation glide. Under applied stress, dislocations must overcome the lattice distortions introduced by oxygen atoms [58], thereby increasing the energy barrier for dislocation movement.

In addition to the solid-solution strengthening provided by oxygen, grain refinement also provides a further contribution to the overall strength of the Ti-15Zr alloy. Generally, metallic materials with finer grain microstructures exhibit higher strength because grain boundaries act as obstacles to dislocation propagation. When grain size is sufficiently small, the relationship between grain size and yield strength can be described by the Hall–Petch equation [59], which quantifies the strengthening effect of grain refinement, indicating that yield strength increases as grain size decreases. The Hall–Petch relationship can be expressed as follows:

(2) $$\sigma_{ys\left(HP\right)}=\sigma_{0}+kd^{-0.5}$$

where $\sigma_{0}$ is the intrinsic yield strength of the material, k is the Hall–Petch constant related to the material, d is the average grain size of the Ti-15Zr-xO alloy.

It is important to clarify the methodology of the strengthening model presented here. The model deconstructs the total yield strength into several key contributions. The experimentally measured yield strength of the baseline Ti-15Zr alloy (619.2 MPa) serves as a crucial reference for this deconstruction. This baseline strength is not that of a pure Ti-Zr alloy, but already incorporates contributions from its initial microstructure and composition. Specifically, this includes the significant solid-solution strengthening effect from the inherent 0.14 wt.% oxygen content, which is determined to be 280.4 MPa based on our analysis. Furthermore, other mechanisms contribute to the overall strength, such as the substitutional solid-solution strengthening from the 15 wt.% zirconium content and the strengthening from the initial grain structure and dislocation density. As the Zr content and the initial processing state are constant across all samples, these contributions are combined into a constant “effective matrix strength” (σ_matrix). By subtracting the independent contributions of the initial oxygen (280.4 MPa) and the initial grain structure (15.1 MPa) from the baseline experimental strength (619.2 MPa), this effective matrix strength is calculated to be 324.1 MPa. Therefore, the quantitative analysis in this study models the total theoretical strength of all alloys by summing the constant matrix strength (324.1 MPa) with the total contributions from grain refinement and the total solid solution strengthening for each specific composition. Since the value of $\sigma_{0}$ for the Ti-15Zr-xO alloy is the same as that of Ti-15Zr, the improvement in the compressive strength of the alloy due to grain refinement strengthening can be expressed as,

(3) $$\Delta \sigma_{ys\left(HP\right)}=k\left(d^{-0.5}-d_{0}^{-0.5}\right)$$

where $d_{0}$ is the average grain size of Ti-15Zr, with its value shown in Figure 9a; the value of k is 22.5 MPa/mm-0.5 [5]. The value of k is taken as 22.5 MPa·mm^0.5^, which is a widely accepted value for Ti-based alloys. It should be noted that this analysis assumes the Hall–Petch coefficient k is a constant. However, it is known that high concentrations of interstitial solutes, such as oxygen, can segregate to grain boundaries and influence their ability to impede dislocation motion, potentially altering the k value. Quantifying this specific effect would require extensive further research and is beyond the scope of this study. Therefore, using a constant, the literature-derived k value is considered a reasonable approximation for this analysis. Given that the overall contribution from grain refinement is found to be modest, any minor variation in k would not alter the primary conclusion that solid-solution strengthening is the dominant mechanism.

At the atomic scale, atoms dissolved in the alloy, whether interstitial or substitutional, can significantly affect the material’s yield strength. This is because the interaction between dislocations and solute atoms increases the obstacles to dislocation motion, leading to an increase in yield strength. This increase is generally proportional to the concentration of solute atoms, reflecting the basic principle of solid solution strengthening. The solid solution strengthening effect can be described by various theoretical models, among which the Labusch model [60] is a widely accepted and representative model, expressed as:

(4) $$\Delta \sigma_{ys\left(SS\right)}=\frac{\tau }{S_{F}}=\frac{c^{2/3}}{S_{F}}{\left(\frac{F_{m}^{4}w}{4Gb^{9}}\right)}^{1/3}$$

where c is the atomic ratio of interstitial atoms, $S_{F}$ is the Schmid factor analyzed from each sintered specimen via EBSD, $F_{m}$ is the maximum interaction force, w is the width of the edge dislocation, G is the shear modulus, b is the Burgers vector.

**Figure 12:**
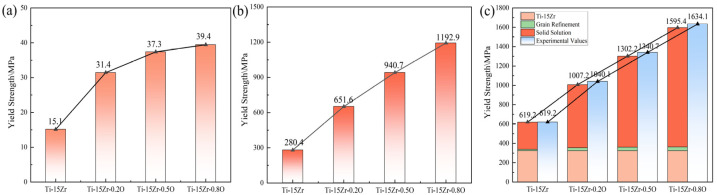
Yield strength contributions from grain refinement and solid solution strengthening: (**a**) grain refinement strengthening; (**b**) solid solution strengthening; (**c**) Combined Yield Strength from Grain Refinement and Solid Solution Strengthening.

The Schmid factor $S_{F}$ can be calculated from EBSD data. Due to the anisotropic strain and stress induced by solute atoms, it is challenging to determine the value of $F_{m}$. Kariya et al. [61] derived the values of $F_{m}$ and $(F_{m}^{4}w/4Gb^{9}{)}^{1/3}$ for oxygen-containing titanium-based materials as 6.22 × 10^−10^ N and 5.3 × 10^3^, respectively, which were then substituted into Equation (4). After the calculation, the values of $(F_{m}^{4}w/4Gb^{9}{)}^{1/3}$ for Ti-15Zr-xO and the linear relationship between the yield strength increment caused by oxygen atoms and are shown in Figure 12. Figure 12a represents the yield strength increment calculated from grain refinement strengthening, while Figure 12b shows the yield strength increment caused by oxygen solution strengthening. Figure 12c displays the theoretical yield strength increment, where the experimental data for Ti-15Zr correspond to the measured values, and the oxygen-induced portion was obtained by adding the calculated grain refinement and oxygen solution strengthening increments. The results were compared with the experimentally measured yield strength.

By directly comparing the calculated contributions, a clear conclusion can be drawn. As shown in Figure 12a, the strength increment from grain refinement strengthening ($\Delta \sigma_{ys\left(HP\right)}$) is quite modest, ranging from approximately 15 to 40 MPa. Concurring with the reviewer’s insight, this contribution is not significant when contrasted with the immense strengthening effect from the solid solution of oxygen ($\Delta \sigma_{ys\left(SS\right)}$), which reaches over 1150 MPa (Figure 12b). In contrast, the solid solution strengthening effect induced by dissolved oxygen atoms is particularly significant, about 650 MPa for Ti-15Zr-0.2O, 940 MPa for Ti-15Zr-0.5O, and 1193 MPa for Ti-15Zr-0.8O. This result demonstrates that the dissolved O atoms effectively enhance the yield strength of the Ti-15Zr alloy. This strengthening effect is primarily attributed to the interaction between oxygen atoms, which act as interstitial atoms, and dislocations, significantly hindering dislocation movement, thereby improving the mechanical properties of the materials.

The quantitative analysis presented in Figure 12 clearly demonstrates a significant disparity between the two primary strengthening mechanisms. The overwhelming contribution to the strength enhancement comes from solid-solution strengthening, while the effect of grain refinement is comparatively modest. This can be understood by considering the underlying physical mechanisms. As a potent interstitial solute, oxygen occupies the octahedral sites within the HCP Ti-lattice, causing severe and highly asymmetric local lattice distortions [62]. These distortions create powerful short-range stress fields that act as extremely effective obstacles to dislocation glide, requiring a significantly higher applied stress to initiate and sustain plastic deformation [63]. In contrast, while grain refinement from 14.5 μm to 9.2 μm provides a beneficial strengthening effect according to the Hall–Petch relationship, the grains remain in the micrometer scale. The strengthening from these grain boundaries is therefore less pronounced than the atomic-scale obstruction caused by the high concentration of interstitial oxygen. This finding underscores that for this class of Ti-Zr-O alloys, precise control of the dissolved oxygen content is the primary and most effective strategy for achieving ultra-high strength, with grain refinement serving as a valuable secondary mechanism.

## 4. Conclusions

The light, economic and innocuous element of oxygen has been added into the Ti-15Zr alloy, successfully preparing the high-strength Ti-Zr-O alloys by spark plasma sintering (SPS) technology. A series of Ti-15Zr alloy samples with varying oxygen contents was prepared by introducing different mass fractions of nano TiO_2_ powder as a means to adjust the oxygen content. The main conclusions are as follows:

(1) XRD analysis confirmed that all sintered samples consist of a single α-Ti phase, indicating that the added TiO_2_ fully decomposed and oxygen dissolved into the Ti-matrix during the SPS process. The SPS technique yielded highly dense samples with relative densities exceeding 99% and significantly reduced porosity. Notably, increasing the oxygen content resulted in a refined, equiaxed α-grain structure with a uniform crystal orientation.
(2) The mechanical tests revealed excellent properties, which are a direct consequence of the dense and fine-grained microstructure fabricated by SPS. The mechanical tests revealed a strong correlation between oxygen content and strength. The compressive yield strength increased linearly from 619.24 MPa in the oxygen-free Ti-15Zr alloy to a remarkable 1634.18 MPa in the Ti-15Zr-0.8O alloy. This substantial strengthening was accompanied by a slight decrease in ductility. Fracture analysis of all samples showed a mixed-mode fracture, characterized by both ductile dimples and brittle cleavage-like features, indicating that the alloys retained a degree of plasticity even at very high strength levels.
(3) Analysis of lattice parameters confirmed that oxygen atoms primarily occupy octahedral interstitial sites within the α-Ti matrix, causing lattice expansion. A quantitative assessment of strengthening mechanisms, based on the Labusch and Hall–Petch models, demonstrated that the observed strength enhancement is predominantly due to the solid solution strengthening effect from dissolved oxygen, while the contribution from grain refinement strengthening was found to be relatively minor.

## Figures and Tables

**Table 1:** Composition of Ti-15Zr metallic powder prepared by gas atomization (wt.%).

Composition	Zr	Fe	C	O	N	H	Ti
Content	14.900	0.012	0.034	0.120	0.006	0.010	Bal.

**Table 2:** Sintering process parameters for experimental specimens.

Sintering Temperature/℃	Sintering Pressure/MPa	Holding Time/Minutes	Heating Rate/°C/Min
1000	100	10	50

**Table 3:** Actual O content of sintered Ti-15Zr samples with different mass fractions of TiO_2_.

Mixed Powder	Sintered Sample
Oxygen Content	Oxygen Content
0.12 wt.%	0.14 wt.%
0.26 wt.%	0.32 wt.%
0.51 wt.%	0.55 wt.%
0.82 wt.%	0.83 wt.%

**Table 4:** Comparison of compressive yield strength and Compression Strain for selected compositions.

Material	Compressive Yield Strength/MPa	Compression Strain/%
Ti–Nb	943	-
Ti-6Al-4V	980	20
Ti–35Nb–5Sn	1120	16
Ti-1.5at%O	1220	15
Ti-13Nb-13Zr	1230	12
Ti-2.0Zr-0.5Ta	1568	34
Ti-15Zr-0.8O	1634	31

**Table 5:** Mechanical properties of sintered Ti-15Zr-xO samples at 1000 °C.

Material	Density/%	Compressive Yield Strength/MPa	Compression Strain/%
Ti-15Zr	99.13	619.2 ± 8.1	50.0 ± 3.3
Ti-15Zr-0.2O	99.27	1040.1 ± 6.5	41.5 ± 1.5
Ti-15Zr-0.5O	99.19	1340.7 ± 5.3	36.8 ± 1.2
Ti-15Zr-0.8O	99.44	1634.1 ± 2.3	31.1 ± 1.4

## Data Availability

The original contributions presented in this study are included in the article. Further inquiries can be directed to the corresponding authors.
